# How Should Community Mental Health of Intellectual Disability Services Evolve?

**DOI:** 10.3390/ijerph110908624

**Published:** 2014-08-25

**Authors:** Colin Hemmings, Nick Bouras, Tom Craig

**Affiliations:** Institute of Psychiatry, Kings College, London WC2R 2LS, UK; E-Mails: Nick.Bouras@kcl.ac.uk (N.B.); thomas.craig@kcl.ac.uk (T.C.)

**Keywords:** community mental health, services, intellectual disabilities

## Abstract

Services for people with Intellectual Disability (ID) and coexisting mental health problems remain undeveloped; research into their effectiveness has been lacking. Three linked recent studies in the UK have provided evidence on essential service provision from staff, service users and carers. Interfaces with mainstream mental health services were seen as problematic: the area of crisis response was seen as a particular problem. Further services’ research is needed, focusing on service components rather than whole service configurations. There was not support for establishing more intensive mental health services for people with ID only. The way forward is in developing new ways of co-working with staff in “mainstream” mental health services. Mental health of ID staff might often be best situated directly within these services.

## 1. Introduction

Services for people with Intellectual Disability (ID) and coexisting mental health (MH) problems remain undeveloped when compared to those for the wider population. Community ID services remain highly variable in constitution and operation. Research into their effectiveness has been lacking [1]. There have been a few studies looking at various services but as they vary so much it has not been easy to compare the findings [2]. It is not clear how best to deliver mental health care within ID services and it would stretch belief to think that all of the differing service configurations and provision would have equal effectiveness and value for money. There have not been the same sorts of service developments seen in recent years in “mainstream” mental health services. Meanwhile mental health services’ research has frequently excluded adults with ID [3].

So although UK Government policy in recent years has promoted the use of mainstream services wherever possible for people with ID and mental health problems (Foundation for People with Learning Disabilities, [4]), this policy itself has not been evidence based. An as yet unanswered question after decades of community care remains, “Do people with ID and mental health problems do better in specialist ID or mainstream MH services?” [5]. Staff, carers and service users have long complained about the misunderstanding and misdiagnosis of people with ID and MH problems and the ongoing problems for them gaining access to mental health services. ID services have often lagged woefully behind even in basics [6].

Accordingly there has been debate about how ID services providing care for people with ID and mental health problems should evolve, particularly in the provision for those at risk of distressing and costly inpatient admissions. There has been interest in more intensive community mental health service models for those with ID [7]. It was questioned whether assertive community treatment (ACT) could be an effective service model for people with ID. This was supported by the finding in the UK-700 study [8] that people with borderline intellectual functioning spent less time in hospital if they received more intensive community care [9]. However the population included in this study were mostly not known to ID services and had a diagnosis of schizophrenic spectrum disorder. A few ACT-type services for people with ID have been set up in the UK, but they have varied widely [7]. Some favoured a “team within a team” model in which clinicians have adopted a more intensive approach whilst being part of a wider community ID team. Other services followed a “separate team” model. Confusingly, some of these services have been aimed at people with ID and challenging behaviours rather than mental illness. There have thus been problems in the evaluation of the various ACT-type models for people with ID [10].

A study of opinions about the ACT model in ID services among specialist ID clinicians found much inconsistency regarding how ACT should be applied [11]. Some saw service users as needing the support of an ACT in ID service as a standalone service endeavouring to provide a range of functions including supporting housing and employment needs; others saw such services as being of a smaller scale that would support access to mainstream mental health care. Some saw the specialist ACT in ID role as consulting to, liaising with and supporting those mainstream services; others felt such a service should endeavour to provide all services seeking additional support or input only if necessary. The findings showed that it would not be possible to research whether adults with ID might benefit from ACT or indeed any other newer mental health service models either when there had been no consensus about what such models should consist of for people with ID nor agreement even of what standard or routine community ID services should provide.

The overall aim of this article is to review recent evidence derived from three linked studies on essential service provision from staff drawn from throughout the UK and service users and carers drawn from one of the longest operating (over 30 years) MH services in the UK.

## 2. What Should Community Services Provide?

Researching the provision of community services for those with ID and mental health problems can be informed by the insights gained from mental health services’ research in the general population [12]. For example it has been argued that individual service components should be researched rather than different service configurations [13]. There has been little investigation into what service users with ID and mental health problems actually want from community services. Carers’ perspectives have been neglected too yet their opinions are often crucial in the care of people with ID [14].

Three linked studies in the UK have investigated what community services should provide for people with ID and mental health problems. The first compared views between specialist ID staff, carers and service users [15]. There were no great differences between the groups as to what they viewed as essential service components. Service users emphasized their need for practical help and for all staff to be trained in ID and to get to know them. Carers emphasized the need for prompt treatment and for more information to be made available, particularly what to do and where to go in a crisis. The clinicians emphasized the need for all involved to have good understanding of both ID and mental health problems. They highlighted interfaces between specialist ID and mainstream mental health services. They wanted to see earlier diagnosis and treatment and access to mainstream services, particularly in a crisis. They were wary of developing new stand-alone services for people with ID and more concerned that existing ID services should be able to respond flexibly and more intensively for people in mental health crises.

In the second study a consensus of opinions was generated among specialist ID clinicians about what service components specialist ID services should provide using the Delphi survey technique [16]. Service components considered essential for routine services included such items as *regular review of service user and care plans*, *monitoring of mental state*, *monitoring of medication*, *crisis plans* and *out of hours support*. Service components considered as essential for more intensive services, included *can react to a crisis that day* and *provide a comprehensive list of contacts should the service user relapse*. It would be invaluable to research further the many service components identified as essential for community services in this study and to evaluate their effective implementation.

In the third study specialist ID clinicians were interviewed in-depth about how some of these key service components should be implemented [17]. Most were “expert” in that they had published research or opinions on community services. Their opinions contained several themes such as the need for services to have clarity of purpose and clear care pathways. They often commented that joint working with mainstream mental health services needed to be greatly improved. Participants frequently talked about the need for all staff to have sufficient training and that specialist ID services needed to provide this. They were concerned that often mainstream mental health services do not meet the needs of people with ID and they were therefore keen to develop new improved ways of joint working.

There was not however support for developing “super-specialist” services within ID services such as ACT. Participants did not believe that there would not be the critical mass of staff or service users or the funding levels to make these economically viable, effective and sustainable, even if these might be theoretically the most ideal. There were concerns that these could increase discontinuity in the already patchy mental health care provided. An opinion repeatedly expressed was that ID services should not seek to provide services that were already available for the wider population, such as a 24-h service.

## 3. Further Community Services’ Research is Needed

Perhaps the ACT model is no longer so relevant to ID services in the UK when other mental health service models such as crisis resolution have become more established [18]. So could these or other newer mental health service delivery models be utilized for people with ID? Owing to the relatively small numbers of service users with ID when compared to the wider population, any whole service (existing or new) evaluation studies are likely to be underpowered or not feasible. Funding for service delivery models not previously evaluated in people with ID is unlikely to be forthcoming. Researchers in ID have historically found it difficult to compete for the huge grants needed for whole service evaluations.

So it is true that we do not know for certain how ID services should evolve as we do not have the evidence base to guide development. But we do know with certainty that mental health service provision for people with ID is lacking in many ways. To break out of this circular problem a more realistic way forward is to look at the modelling, piloting and the evaluation of specific service components, which in themselves can often be considered complex interventions [19]. It will be more feasible for example to undertake studies looking at a specific service component, such as the siting of specialist ID staff within home treatment or crisis response teams. A wider range of outcomes than hospital admissions needs to be investigated in this necessary research [1]. Randomized controlled trials of service provision are certainly crucial to strengthen the evidence base but they need to be given a proper foundation. Often this will necessitate evidence from study designs further back in the research continuum, including qualitative research [10].

Other examples of community service provision which are important to research would be the most effective ways of monitoring mental state and medication adherence and promoting continuing contact with service users with ID prone to disengagement. Important research questions include: What are the interventions or components of service delivery that alter the rates of inpatient admissions? What factors might be currently promoting or obstructing the access of people with ID to newer service models of mental health services? Further research is needed also into the mental health of ID training of staff in mainstream mental health and specialist ID services and also for carers [20]. We also need further research such as that by [21] into psycho-education for service users, carers and families. It would be important to investigate views and actions of staff in mental health and primary care services, and others often involved such as social workers, police and paramedics about their encounters with people with ID and mental health problems. This might also in turn clarify what training might be helpful for them at crucial junctions in the pathways of care.

There is now a consensus among clinicians of the need for crisis services but simple data of this need is often lacking. How often are mental health crises occurring in this service group? How often people with ID in mental health crises are presenting to services? How often mental health inpatient admissions are required? How frequent are presentations of people with ID in mental health crises to a range of settings, including accident and emergency services, police, mental health and specialist ID services? Lunsky *et al.* [22] have reported problems of accessing and using emergency mental health services for people with ID. One example of a new service component that could lead to potential improvements in the interactions between non-ID trained staff and service users with ID and the care provided is the use of personalised accessible crisis information. A recent feasibility study has shown that crisis information can be modified to be made meaningful and valued by people with ID and their carers [23].

## 4. How Should Community Mental Health of Intellectual Disability Services Evolve?

Specialist ID clinicians in the UK do not see the future of their services as replicating service developments in mainstream mental health. There is not widespread support for establishing newer or more intensive mental health services for people with ID only. It seems instead that the best way forward is in developing new and closer ways of joint working with staff in mainstream mental health services. Doubtless there will be many specialist ID clinicians who would argue that their working relationships already show close and effective collaboration. However joint or co-working service provision should not be overly dependent on local relations between ever-changing personnel or based on goodwill only but needs to be protocol and evidence-based and facilitated and not hindered by organisational structures.

Currently few staff in community ID teams are trained in or focus on mental health. With the long history of abuses of people with ID in institutional care there can often be overcompensation from specialist ID staff leading to subtle or even overt criticisms of mental health services. There can often be a stigmatizing attitude towards mental health staff and services from specialist ID staff [7]. This is an ironic kind of reverse stigmatization given the marginalisation of people with ID. Even the widely used term “mainstream” has been noted to be potentially used in a sometimes pejorative way; very often there are assumptions in community ID teams that the ongoing problems for people with ID accessing mental health services lies only with those services [24]. It is important that staff in specialist ID services do not just complain from the side-lines about people with ID being misunderstood or neglected or stigmatized (or themselves by proxy) by mental health services but instead take an active role in assisting these services and not just default to a “they must be trained” mantra. More empathy is sometimes needed by specialist ID staff with a realization that mental health staff have a wide range of co-morbidities and service difficulties to contend with and that the care of people with ID, despite their disproportionate mental health needs, will never be their first priority given their relatively few numbers.

The future of mental health of ID (MHID) services lies directly within “mainstream” mental health services. The best opportunity for improving the mental health care of people with ID may be in seeking to work directly from within mainstream mental health services including the newer crisis, home treatment and “assertive outreach” teams. It might mean that MHID staff are employed directly within these other mental health services. MHID staff would then be highly visible, showing what they can “bring to the table” to assist hard-pressed mental health colleagues [25]. MHID staff would concentrate on a core group with ID who need continuing mental health care, high support and more intensive input at times. The other main function of MHID staff should be to enhance the mental health assessments of people with low IQ and a range of complex needs such as mental illness, autism, personality disorders and/or epilepsy. This may mean being working with some people on the “other side” of the IQ boundary that would benefit from specialist MHID input [25].

## 5. Conclusions

Service provision for those dually disadvantaged by ID and coexisting mental health problems is still heavily determined by widespread and enduring dualistic thinking. This leaves it bounced between mental health and ID services’ commissioning. The staff employed in MHID services need to advise commissioners where they will be most likely to be most effectively located. It may be that in some areas MHID staff, in order to align more closely with their mental health colleagues, split organisationally from other specialist ID health services, including those staff primarily concerned with challenging behaviour without diagnosable mental illness. MHID service provision could then become more clearly focused, integrated, supported and hopefully, more effective.
